# Myocardial susceptibility to ischaemia/reperfusion in obesity: a re-evaluation of the effects of age

**DOI:** 10.1186/s12899-017-0030-y

**Published:** 2017-03-17

**Authors:** I. Webster, R. Salie, E. Marais, W.-J. Fan, G. Maarman, B. Huisamen, A. Lochner

**Affiliations:** 1 Division of Medical Physiology, Department of Biomedical Sciences, Faculty of Medicine and Health Sciences, University of Stellenbosch, Stellenbosch, South Africa; 2 Biotechnology and Innovation Platform of the SA Medical Research Council, Cape Town, South Africa

**Keywords:** Age, Diet-induced obesity, Ischaemic preconditioning, Infarct size, Myocardial function

## Abstract

**Background:**

Reports on the effect of age and obesity on myocardial ischaemia/reperfusion (I/R) injury and ischaemic preconditioning are contradictory. The aim of this study was to re-evaluate the effects of age and diet-induced obesity (DIO) on myocardial I/R injury and preconditioning potential.

**Methods:**

Four groups of Wistar male rats were used: age-matched controls (AMC) receiving standard rat chow for (i) 16 weeks and (ii) 16 months respectively; DIO rats receiving a sucrose-supplemented diet for (iii) 16 weeks and (iv) 16 months respectively. The ages of groups (i) and (iii) were 22 weeks (“young”) and groups (ii) and (iv) 17 months (“middle-aged”) at time of experimentation. Isolated perfused working hearts were subjected to 35 min regional ischaemia/1 h reperfusion. Endpoints were infarct size (tetrazolium staining) and functional recovery. Hearts were preconditioned by 3 × 5 min ischaemia/5 min reperfusion. Results were processed using GraphPad Prism statistical software.

**Results:**

Age did not affect baseline heart function before induction of ischaemia and I/R damage as indicated by infarct size and similar values were obtained in hearts from both age groups. Age also had no effect on functional recovery of hearts during reperfusion after regional ischaemia in AMC rats, but cardiac output during reperfusion was better in hearts from middle-aged than young DIO rats.

The diet reduced infarct size in hearts from young rats (% of area at risk: AMC: 32.4 ± 3.6; DIO: 20.7 ± 2.9, *p* < 0.05), with no differences in hearts from middle-aged rats (AMC: 24.6 ± 4.6; DIO: 28.3 ± 13.5, *p* = NS). Compared to their respective AMC, diet-induced obesity had no significant effect on functional recovery of hearts from both age groups after exposure to regional ischaemia.

When exposed to the more severe stress of global ischaemia, the functional recovery potential of middle-aged DIO rats appeared to be impeded compared to hearts of young DIO rats, while age had no effect on the functional recovery of AMC hearts.

Preconditioning reduced infarct size in hearts from young control rats and both middle-aged groups, but not from young DIO rats. Age had a significant effect on functional recovery in preconditioning: it was improved in hearts from young control and DIO rats, but depressed in both middle-aged groups.

**Conclusions:**

The data showed that middle-age and obesity had no effect on baseline myocardial function and did not increase susceptibility to I/R damage upon exposure to regional ischaemia. On the contrary, obesity reduced I/R damage in young rats. Preconditioned aging hearts showed a decreased infarct size, but a reduction in functional recovery.

## Background

The effect of ischaemia/reperfusion (I/R) on the myocardium as well as its response to cardioprotective interventions are influenced by several variables, amongst others, age and obesity. The respective contributions of the latter two factors have recently been extensively reviewed [1–4].

It is of significance that the vast majority of studies on the response of the heart to I/R injury, the processes involved and the consequences of interventions, have been conducted on healthy or mature populations of experimental animals. As cardiovascular diseases are the leading cause of death, particularly in the elderly, cognizance should be taken of the role of age-induced changes in the response of the heart to pathophysiological conditions. The effects of increasing age on the cardiovascular system are well-established and entail, amongst others, an increase in left ventricular mass, myocyte hypertrophy and a reduction in myocyte number [5–7]. Apart from the significant changes in β-adrenergic signalling [8, 9], mitochondrial ROS production also increases with age, which in turn, may cause mitochondrial damage [6]. The amounts of ROS generated are critical for the fate of the cell, since low amounts can function as signalling molecules and are important in cardioprotection, whereas high amounts are detrimental by opening the mitochondrial permeability transition pore, causing cell death [10].

Aging decreases the intrinsic tolerance of the heart to ischaemic injury. For example, it has been shown that loss of myocardial tolerance to ischaemia in the mouse begins during the middle-age (~12 months) and becomes outspoken at 18–24 months of age [11]. This increased susceptibility of the heart to I/R damage is likely to be the consequence of enhanced oxidative stress [11–13].

In addition to the effects of old age on the response of the heart to I/R, obesity is also considered to be a serious risk factor in the development of cardiovascular disorders and is regarded to impact negatively on the outcome of myocardial ischaemia. Since obesity associated with insulin resistance has been demonstrated to increase oxidative stress, and is associated with coronary artery disease [14–16], I/R occurring in this condition could provoke a further increase in oxidative stress. However experimental studies reported a variety of outcomes. A decreased myocardial tolerance to I/R damage was observed in vivo [17] and ex vivo studies using hyperphagia-induced obese insulin resistant male Wistar rats [18–21]. These studies have shown that an increased infarct size is associated with concomitant poor functional recovery in hearts from obese rats compared with controls. In contrast to the above, smaller infarcts and an improved functional recovery during reperfusion were reported by Donner and coworkers [22] also using a hyperphagia-induced obesity rat model. A recent study from our own laboratory also showed that hearts from rats fed either a sucrose-supplemented diet or a high fat diet for 16 weeks presented with a significant reduction in infarct size when subjected to 35 min regional ischaemia/60 min reperfusion, when compared to age-matched controls [23]. Furthermore, the degree of left ventricular (LV) dysfunction in Zucker obese rats during I/R was reported to be similar to that of lean rats [24]. At the moment the reason(s) for these discrepancies are not clear and could be another manifestation of the phenomenon of the “obesity-paradox” which has been observed in humans: Observational studies have shown that obese heart failure patients tend to have higher survival rates compared to patients with heart failure and normal BMI values [25, 26].

In agreement with the marked effects of age on the susceptibility of the heart to I/R damage, a number of experimental studies showed that the cardioprotective power of ischaemic pre- and postconditioning waned with ageing (for reviews see [1–3]), despite variations in age and preconditioning protocols. The majority of these studies used haemodynamic recovery as endpoint [27–30], with only a few employing the more robust endpoint of infarct size [31, 32]. . It should be borne in mind that in these ex vivo studies, using the isolated perfused heart, the period of ischaemia was followed by immediate reperfusion, usually for 60 min, during which time functional recovery was determined. This protocol therefore does not allow evaluation of chronic recovery. Clearly the effects of age and obesity should also be evaluated in an in vivo model which allows study of the consequences of I/R on chronic recovery.

In view of earlier observations made in our laboratory, namely that hearts from rats with hyperphagia-induced insulin-resistance, aged 22 weeks, are characterized by an unaltered functional performance and an improvement in resistance to I/R injury [23], the aim of the present study was to re-evaluate the effects of age and obesity-induced insulin resistance on myocardial susceptibility to I/R damage and functional performance during reperfusion as well as the capacity to be protected by prior ischaemic preconditioning. For this purpose two age groups were used, namely 22 weeks and 17 months. The precise correlation between the age of laboratory rats and the human is still a matter of debate and complicated by the different phases of life. It has been estimated that in general, considering their entire life span, a human month resembles one every-day life of a laboratory rat [33]. Based on these assumptions, it was calculated that the 22 weeks old and 17 months old rats represent 13 and 43 human years respectively and were referred to as “young” and “middle-aged” throughout the article.

## Methods

### Animals

Age and weight matched male Wistar rats were used in this study. This study was approved by the Committee for Ethical Animal Research of the Faculty of Medicine and Health Sciences, University of Stellenbosch (ethical approval number SU-ACUM11-00002). Animals were treated according to the revised South African National Standard for the Care and Use of Animals for Scientific Purposes (South African Bureau of Standards, SANS 10386, 2008). Rats were obtained from the University of Stellenbosch Central Research Facility. Two to three rats were housed in one cage. They received water and food ad libitum (light/dark cycle 6 h00–18 h00; temperature 22 °C; humidity 40%). Rats aged 6 weeks were divided into four groups: (i) age matched control (AMC) rats received a standard commercial rat chow for 16 weeks, (ii) diet-induced obese rats received a sucrose-supplemented diet (DIO) for 16 weeks, (iii) age-matched control rats received standard rat chow for 16 months and (iv) diet-induced obese rats received the DIO diet for 16 months The age of groups (i) and (ii) at the time of experimentation averaged 22 weeks (“young”), while the age of groups (iii) and (iv) averaged 17 months (middle-aged) at the time of experimentation. Except in the case of the echographic studies where four rats per group were studied, in all other experiments the *n* values varied between 6 and 9 rats/group. Based on previous experimentation in our laboratory, these numbers were sufficient for the purpose of the study [20, 21]. The DIO diet was prepared by addition of condensed milk and sucrose to the standard rat chow as described by Pickavance and coworkers [34]. The composition of the control and DIO diets was analyzed by Microchem, Cape Town: the DIO diet contained three and four fold more saturated fats and sucrose respectively than the control diet while the total carbohydrate content was 33% higher, as described before [23].

### Chemicals

Routine chemicals (pro analysi) were obtained from Merck Chemical Co (Cape Town, RSA).

### Experimental procedure

At the end of the feeding period, non-fasted rats were weighed and anaesthetized with pentobarbital (30 mg/rat). They were then sacrificed, the hearts removed and arrested in ice-cold saline for subsequent perfusion in the working mode. The visceral fat was collected and weighed.

### Perfusion technique

Hearts were perfused as described before [35]. Briefly, a modified Krebs-Henseleit bicarbonate buffer was used, containing (in mM): NaCl 119; NaHCO_3_ 24.9; KCl 4.7; KH_2_PO_4_ 1.2; MgSO_4_.7H_2_O 0.59; Na_2_SO_4_ 0.59; CaCl_2_ 1.25; glucose 10. The buffer was oxygenated with a 95%oxygen/5%CO_2_ gas mixture at 37 °C.

All perfused hearts were stabilized by retrograde perfusion for 15 min, followed by perfusion for 15 min in the working mode (preload 15 cm H_2_O, afterload 100 cm H_2_O), during which time the aortic and coronary flow rates were measured. Systolic pressure and heart rate were measured through a side-arm of the aortic cannula connected to a Viggo-Spectramed pressure transducer coupled to a computer system.

After stabilization, hearts were perfused for 30 min in the retrograde mode, subjected to 15 min global ischaemia or 35 min regional ischaemia (36.5 °C) and 60 min reperfusion for measurements of functional recovery (at 20 and 30 min reperfusion) and infarct size after 60 min. The ability to be preconditioned was studied in DIO and age-matched control rats of both age groups. In these studies, after the 30 min stabilization period, hearts were perfused retrogradely for either 30 min (non-preconditioned (NPC)) or for 10 min followed by a preconditioning protocol of 3x5min ischaemia/5 min reperfusion, followed by 35 min regional ischaemia (36.5 °C) and 60 min reperfusion for measurements of functional recovery and infarct size.

### Determination of infarct size

Myocardial infarct size was determined as described previously, using tetrazolium staining [36]. Each heart was cut in 2 mm thick slices. For each slice the left ventricle area at risk and the infarcted areas were determined using computerized planimetry (UTHCSA Image Tool programme, University of Texas Health Science Center at San Antonio, TX, USA).

### Echocardiography

Echocardiography was used to monitor the heart function and structure in vivo. Cardiac geometry and function were evaluated in anaesthetized (2.0% Isoflurane) rats using two-dimensional guided M-mode echocardiography (Vivid e, GE Healthcare) equipped with a 15–2 MHz linear transducer. Wall thicknesses and diastolic and systolic left ventricular dimensions were recorded from M-mode images acquired through the anterior and posterior LV walls at the papillary muscle level. This was a blinded study, and all studies were performed and interpreted by an independent operator.

### Statistical analysis

Results were processed using GraphPad Prism statistical software (versions 5 and 6). All results were expressed as mean ± standard error of the mean (SEM). For comparisons between groups, an analysis of variance (ANOVA), followed by the Bonferroni correction was applied. Comparison between functional performance before and after ischaemia during reperfusion, was done by use of Student’s *t* test. A *p*-value of *p* < 0.05 was considered significant.

The manuscript has been prepared according to the ARRIVE guidelines (http://www.nc3rs.org.uk/page.asp?id=1357), using the ARRIVE Guidelines checklist.

## Results

### Biometric data (Table 1)

**Table 1:** Biometric data: effects of age and diet

	Body weight (g)	Intraperitoneal fat (g)
22 weeks		
Age-matched controls (22)	362 ± 8.8	10.7 ± 0.9
DIO (30)	469 ± 7.9^*^	24.7 ± 1.0^*^
17 months		
Age-matched controls (21)	682 ± 16	64.4 ± 3.5
DIO (23)	836 ± 51^*^	93 ± 5.8^*^

Values in brackets indicate number of animals

^*^ *p* < 0.05 vs Age-matched controls

The body mass of rats on the DIO diet was significantly higher than those of their age-matched controls in both young (22 weeks) (30% higher) and middle-aged (17 months) (23% higher) groups. Similarly in both groups the intraperitoneal fat was significantly higher in rats on the DIO diet. However, the % change in the latter parameter was significantly higher in the young diet group (131%), than in the middle-aged (44%) rats, when compared to their respective age-matched controls.

### Infarct size

**Fig. 1:**
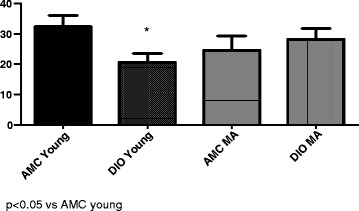
Effect of age and diet on infarct size (% of area at risk). *n* = 6/group. Abbreviations: AMC: age-matched controls: DIO: diet-induced obese rats; MA: middle-aged

Age did not appear to affect the susceptibility of the heart to I/R damage: there was no significant difference in infarct size of hearts from young and middle-aged age-matched control rats when subjected to the same period of regional ischaemia (35 min) followed by 1 h reperfusion. Similarly there were no significant differences between infarct sizes of hearts from young and middle-aged DIO rats when exposed to a similar period of regional ischaemia and reperfusion (Fig. 1).

As was also observed in an earlier study from our laboratory [23], the DIO diet for 22 weeks had a significant effect on infarct size after 35 min coronary artery ligation: infarct size was significantly lower in hearts from young rats when compared with their age-matched controls (Infarct size, % of area at risk: AMC 32.4 ± 3.6; DIO 20.7 ± 2.9; *p* < 0.05). Interestingly, in hearts from middle-aged rats no differences in infarct size were observed between the control and DIO groups (AMC: 24.6 ± 4.6; DIO 28.3 ± 3.5) (Fig. 1).

The area at risk did not differ between the DIO and age-matched controls in both age groups (young rats: AMC: 51.42 ± 1.33; DIO: 53.41 ± 0.87; middle-aged rats: AMC: 53.28 ± 4.5; DIO: 49.64 ± 5.0).

### Functional recovery during reperfusion after 35 min regional ischaemia

For evaluation of the function of the heart under baseline conditions and its response to ischaemia and reperfusion, use was made of the working rat heart model which allows measurement of cardiac pump function at fixed pre- and afterloads [37]. Failure to produce aortic output during reperfusion, while still capable of generating pressure (as reflected by the peak systolic pressure development), has a significant effect on the evaluation of Total Work performed. We therefore also indicated the number of hearts able to produce aortic output during reperfusion (Tables 2, 3, 4 and 5), as this is also regarded as an additional indicator of the recovery potential of the heart during reperfusion.

**Table 2:** Effect of age and diet on functional recovery during reperfusion after 35 min regional ischaemia

		Coronary flow (ml/min)	Aortic flow (ml/min)	Cardiac output (ml/min)	Heart rate (beats/min)	PSP (mm Hg)	Total Work (mW)	Total work (% recovery)
Age 22 weeks								
Age-matched controls (6)								
^a^3/6	Before	18.3 ± 0.6	39.7 ± 3.5	57.7 ± 3.8	259 ± 6	92 ± 2	11.86 ± 1.01	
	After	6.9 ± 3.1	8.7 ± 4.1	15.6 ± 7.1	127 ± 57	42 ± 19	2.64 ± 1.23	19.7 ± 9.4
	*p*	0.0051	0.0002	0.0004	0.0422	0.0226	0.0002	
DIO (6)								
^a^6/6	Before	17.3 ± 0.7	33.7 ± 0.8	51.0 ± 0.9	243 ± 8	92 ± 1	10.48 ± 0.32	
	After	10.4 ± 1.0	9.2 ± 1.6	19.5 ± 1.7	246 ± 9	84 ± 1.9	3.67 ± 0.38	34.8 ± 2.99^b^
	*P*	0.0002	0.0001	0.0001	NS	0.0134	0.0001	
Age 17 months								
Age-matched controls (8)								
^a^5/8	Before	20.5 ± 1.5	45.0 ± 1.4	65.5 ± 2.0	235 ± 11	104 ± 1	15.02 ± 0.50	
	After	22.1 ± 2.4	13.6 ± 4.9	35.8 ± 6.3	236 ± 37	77 ± 11	6.58 ± 1.58	42.4 ± 9.2
	*p*	NS	0.0001	0.0005	NS	0.0409	0.0003	
	p1	0.0021	NS	NS	NS	NS	NS	NS
DIO (9)								
^a^7/9	Before	19.0 ± 21	37.6 ± 3.4	56.6 ± 4.0	253 ± 17	100 ± 3	12.62 ± 1.12	
	After	22.3 ± 2.6	7.2 ± 1.8	29.6 ± 3.2	211 ± 34	77 ± 10	5.24 ± 0.86	41.76 ± 6.6^b^
	*p*	NS	0.0001	0.0003	NS	0.0375	0.0005	
	p2	0.0037	NS	0.0309	NS	NS	NS	NS

*p* values indicate significance of difference between “after” and “before” values in each group

p1 indicate significance of difference between “after” values of young and middle- aged matched controls

p2 values indicate significance of difference between “after” values of young and middle-aged DIO

Numbers in brackets indicate number of rats

^a^ Indicates number of hearts able to produce aortic output during reperfusion

^b^ % recovery in Total work did not differ significantly from age-matched control

**Table 3:** Effect of age and diet on functional recovery during reperfusion after 15 min global ischaemia

		Coronary flow (ml/min)	Aortic flow (ml/min)	Cardiac output (ml/min)	Heart rate (beats/min)	PSP (mm Hg)	Total Work (mW)	Total work (% recovery)
22 Weeks								
Age-Matched Controls (6)								
^a^6/6	Before	18.5 ± 0.9	46.1 ± 1.7	64.6 ± 2.2	282 ± 9	100 ± 2	14.40 ± 0.50	
	After	15.5 ± 1.4	23.1 ± 3.2	38.7 ± 4.1	277 ± 9	90 ± 2	7.81 ± 0.90	53.1 ± 4.8
	*p*	NS	0.005	0.0001	NS	0.0001	0.0001	
DIO (6)								
^a^6/6	Before	19.1 ± 1.2	43.2 ± 1.3	62.3 ± 2.1	285 ± 9	99 ± 1	13.80 ± 0.47	69.4 ± 3.0+
	After	17.7 ± 0.8	28.4 ± 1.2	46.1 ± 1.5	272 ± 7	91 ± 1	9.38 ± 0.40	
	*p*	NS	0.0001	0.0001	NS	0.001	0.0001	
	p1	NS	NS	NS	NS	NS	NS	
17 months								
Age-matched controls (6)								
^a^5/6	Before	19.0 ± 3	48.0 ± 3.0	67.0 ± 3.6	274 ± 11	101 ± 2	14.91 ± 0.9	
	After	19.0 ± 1	29.7 ± 8.1	48.7 ± 8.7	223 ± 47	80 ± 16	9.72 ± 2.47	66.8 ± 16.7
	*p*	NS	0.041	NS	NS	NS	NS	
	p2	NS	NS	NS	NS	NS	NS	NS
DIO (6)								
^a^3/6	Before	21.7 ± 1.7	36.3 ± 6.5	58.0 ± 6.5	258 ± 8	99 ± 2.5	12.84 ± 1.71	
	After	21.3 ± 1.7	18.0 ± 9.3	39.3 ± 9.4	107 ± 53	47 ± 21	6.21 ± 3.0	43.6 ± 20.1
	*p*	NS	NS	NS	0.026	NS	NS	NS
	p1	NS	NS	NS	0.05	NS	NS	NS
	p3	0.049	NS	NS	0.001	0.0172	NS	NS

*p* values indicate significance of difference between “before” and “after” values in each group; p1 values indicate significance of difference between “after” values of age-matched controls and DIO at 22 weeks and 17 months respectively; p2 values indicate significance of difference between “after” values of controls at 22 weeks and 17 months; p3 values indicate significance of difference between “after” values of DIO at 22 weeks and 17 months. ^a^ Number of hearts which produced aortic output during reperfusion. Number in brackets indicate number of rats. + *p* = 0.0086 vs 22 weeks age-matched controls

**Table 4:** Effect of ischaemic preconditioning on functional recovery during reperfusion after 35 min regional ischaemia in young (22 weeks) rats

		Coronary flow (ml/min)	Aortic flow (ml/min)	Cardiac output (ml/min)	Heart rate (beats/min)	PSP (mm Hg)	Total Work (mW)	Total work (% recovery)
Age-matched controls								
Non-preconditioned (6)								
^a^3/6	Before	18.3 ± 0.6	39.7 ± 3.5	57.7 ± 3.8	259 ± 6	92 ± 2	11.86 ± 1.01	
	After	6.9 ± 3.1	8.7 ± 4.1	15.6 ± 7.1	127 ± 57	42. ± 19	2.64 ± 1.23	19.7 ± 9.4
	*p*	0.0051	0.0002	0.0004	0.0422	0.0226	0.0002	
Preconditioned (5)								
^a^5/5	Before	20.8 ± 0.8	37.2 ± 1.2	58 ± 1.8	261 ± 22	91 ± 3	12.2 ± 0.64	
	After	15.2 ± 0.8	23.2 ± 2.3	38.4 ± 3.0	243 ± 17	83 ± 2	7.49 ± 0.86	60.8 ± 4.5
	*p*	0.0046	0.0008	0.0009	NS	0.0072	0.0004	
	p1	0.0441	0.0166	0.0226	NS	NS	0.0124	0.0051
DIO								
Non-preconditioned (6)								
^a^6/6	Before	17.3 ± 0.7	33.7 ± 0.5	51.0 ± 10.9	243 ± 8	92 ± 1	10.48 ± 0.32	
	After	10.4 ± 1	9.2 ± 1.6	19.5 ± 1.7	246 ± 9	84 ± 1.9	3.67 ± 0.38	34.8 ± 2.99
	*p*	0.0002	0.0001	0.0001	NS	0.0134	0.0001	
Preconditioned (5)								
^a^4/5	Before	20.8 ± 1.5	37.6 ± 3.0	58.4 ± 3.7	255 ± 6	94 ± 4	12.3 ± 0.89	
	After	15.2 ± 4.0	20.0 ± 5.2	35.2 ± 8.9	211 ± 53	71 ± 18	7.07 ± 1.78	54 ± 13
	*p*	NS	NS	0.0056	NS	NS	0.0091	
	p2	NS	NS	NS	NS	NS	NS	NS

*p* values indicate significance of difference between “after” and “before” values in each group

p1 values indicate significance of difference between “after” function of non-preconditioned and preconditioned hearts from age-matched control rats

p2 values indicate significance of difference between “after” function of non-preconditioned and preconditioned hearts from DIO rats

Numbers in brackets indicate number of rats

^a^ Indicate number of hearts able to produce aortic output during reperfusion

**Table 5:** Effect of ischaemic preconditioning on functional recovery during reperfusion after 35 min regional ischaemia in middle-aged (17 months) rats

		Coronary flow (ml/min)	Aortic flow (ml/min)	Cardiac output (ml/min)	Heart rate (beats/min)	PSP (mm Hg)	Total Work (mW)	Total work (% recovery)
Age-matched controls								
Non-preconditioned (8)								
^a^7/8	Before	20.5 ± 1.5	45.0 ± 1.4	65.5 ± 2.0	235 ± 11	104 ± 1	15.02 ± 0.5	
	After	22.1 ± 2.4	13.6 ± 4.9	35.8 ± 6.3	236 ± 37	77 ± 11	6.58 ± 1.58	42.4 ± 9.2
	*p*	NS	0.0001	0.0005	NS	0.0409	0.0003	
Preconditioned (6)								
^a^2/6	Before	18.5 ± 2.9	26.8 ± 5.5	45.3 ± 7.3	216 ± 27	104 ± 7	10.29 ± 1.62	
	After	12.0 ± 5.6	6.0 ± 4.1	18.0 ± 8.8	164 ± 56	51 ± 17	3.38 ± 1.78	28.4 ± 12.1
	*P*	NS	0.0123	0.0372	NS	0.0163	0.0168	
	p1	NS	NS	NS	NS	NS	NS	NS
DIO								
Non-preconditioned (9)								
^a^8/9	Before	19.0 ± 2.1	37.6 ± 3.4	56.6 ± 4	253 ± 17	100 ± 3	12.62 ± 1.12	
	After	22.3 ± 2.6	7.2 ± 1.8	29.6 ± 3.2	211 ± 34	77 ± 10	5.24 ± 0.86	41.8 ± 6.6
	*p*	NS	0.0001	0.0003	NS	0.0375	0.0005	
Preconditioned (6)								
^a^1/6	Before	17.8 ± 1.64	26.2 ± 5.6	44.0 ± 6.6	232 ± 32	108 ± 9	10.43 ± 1.8	
	After	15.0 ± 4.8	0	15.0 ± 4.8	164 ± 53	45 ± 15	1.81 ± 0.26	21.6 ± 7.9
	*p*	NS	0.0009	0.0056	NS	0.0065	0.0011	
	p2	NS	0.0078	0.0218	NS	NS	0.0114	NS

*p* values indicate significance of difference between “after” and “before” values in each group

p1 values indicate significance of difference between “after” function of non-preconditioned and preconditioned hearts from age-matched control rats

p2 values indicate significance of difference between “after” function of non-preconditioned and preconditioned hearts from DIO rats

^a^ Indicates number of hearts producing aortic output during reperfusion

Interestingly, function of the isolated working heart *before* induction of ischaemia, was not affected by age and similar values were obtained in hearts from young and middle-aged age-matched control as well as DIO rats. At both ages studied, in all four groups, functional recovery during reperfusion, was significantly lower than that observed during the stabilization phase before induction of ischaemia, with only a few exceptions namely unchanged heart rates in hearts from young and middle-aged DIO rats as well as in hearts from middle-aged controls, while coronary flow rates during reperfusion of middle-aged control and DIO hearts were also unaltered (see Table 2).

However, although the changes were of borderline significance, middle-aged control hearts appeared to recover better during reperfusion (5/8) than hearts from their young counterparts (3/6). In particular, while coronary flow during reperfusion was significantly lower in hearts from young rats (62% reduction), no change in this parameter was observed in hearts from middle-aged rats. This improved performance was also reflected by the higher percentage recovery in Total Work performed (young: 19.7 ± 9.4 vs middle-aged: 42.4 ± 9.2).

In hearts from DIO rats, age had less effect on functional recovery despite the lower coronary flow rate during reperfusion of hearts from young rats, compared to no change in the middle-aged group. The percentage recovery in Total Work performed averaged 34.8 ± 2.99% in young hearts compared to 41.8 ± 6.6% in hearts from middle-aged animals, while 6/6 and 8/9 hearts were able to produce aortic output during reperfusion in the young and middle-aged DIO groups respectively.

The diet had very little effect on functional recovery. In the case of hearts from the young group, all parameters during reperfusion (except heart rate) were similar in hearts from age-matched controls and DIO rats. In addition, the percentage recovery in the Total Work did not differ significantly between the groups (age-matched control hearts 19.7 ± 9.4%, vs DIO 34.8 ± 3.0%, p NS). However, it does appear that at this age, hearts from the DIO group were able to withstand a period of ischaemia better than their control age-matched counterparts since all these hearts were able to produce aortic output (6/6), compared to 3/6 in the control group.

In the case of the middle-aged rats, the diet was also without effect on functional recovery and similar values were obtained in hearts from control and DIO rats during reperfusion. The percentage recovery in Total Work averaged 42.4 ± 9.2% and 41.8 ± 6.6% in hearts from control and DIO rats respectively.

### Functional recovery of hearts subjected to 15 min global ischaemia

To further evaluate the effect of age and diet on the susceptibility of the heart to I/R damage, hearts from young and middle-aged rats were subjected to a more severe stress namely 15 min global ischaemia and functional recovery during reperfusion determined (Table 3). As was observed after regional ischaemia, exposure of hearts from young control and DIO rats to 15 min global ischaemia caused a highly significant reduction in aortic output, cardiac output, PSP and Total Work. This reduction in functional performance was much less in both control and DIO hearts at 17 months of age. Although recovery of hearts from young and middle-aged control hearts was similar, the data obtained suggest that hearts from middle-aged DIO rats were more susceptible to severe ischaemia: although the percentage recovery in Total Work during reperfusion of the older groups did not differ significantly (control 66.8 ± 16.7% vs DIO 43.6 ± 20.11%, *p* > 0.05), 5/6 of the control hearts produced aortic output during reperfusion, compared to 3/6 in the diet group. In addition, the heart rate and PSP of the latter group were significantly lower than those of their younger DIO counterparts.

### Effect of age and diet on ischaemic preconditioning potential

**Fig. 2:**
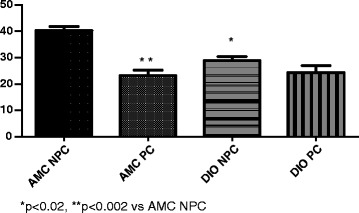
Effect of ischaemic preconditioning on infarct size (% of area at risk). Young (22 weeks) rats. *n* = 6–8/group. Abbreviations: AMC: age-matched controls; DIO: diet-induced obese rats; NPC: non-preconditioned; PC: preconditioned

As previously reported from our laboratory [23], hearts from young control rats can be preconditioned effectively by exposure to a preconditioning protocol of 3 × 5 min ischaemia/reperfusion: this resulted in a highly significant reduction in infarct size (*P* < 0.002). At this age infarct size of NPC DIO hearts was significantly lower than those of NPC hearts from age-matched controls (*p* < 0.02). These hearts could not be further protected against ischaemic damage by prior preconditioning and infarct size was similar to those of NPC DIO hearts (Fig. 2).

**Fig. 3:**
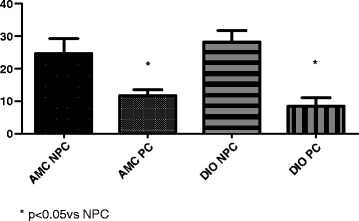
Effect of ischaemic preconditioning on infarct size (% of area at risk). Middle-aged (M-A) (17 months) rats. *n* = 6–8/group. Abbreviations: See Fig. 2

After 17 months on the diet, the pattern changed (Fig. 3): at this age, hearts from control rats as well as hearts from DIO rats could be effectively preconditioned causing a significant reduction in infarct size in both groups.

The areas at risk for hearts from age matched control and DIO rats (NPC as well as preconditioned) did not differ (results not shown).

### Ischaemic preconditioning and functional recovery during reperfusion

As previously observed (Table 2), no significant difference was observed in the baseline function before the onset of ischaemia of NPC as well as PC hearts from young control age-matched and DIO rats (see Table 4). In the NPC hearts from both control and DIO groups, exposure to 35 min regional ischaemia caused a significant reduction in coronary flow, aortic flow, cardiac output, PSP and total work during reperfusion, compared to values obtained before induction of ischaemia.

Ischaemic preconditioning caused a significant improvement in several parameters of function during reperfusion in hearts from young control rats (Table 4). This was particularly evident in the case of coronary flow, aortic flow, cardiac output and Total Work performed during reperfusion. For example, preconditioning increased the percentage recovery in Total Work from 19.7 ± 9.4 to 60.8 ± 4.5 (*p* = 0.0051) in these hearts. This improvement in post-ischaemic outcome was also seen in the fact that all (5/5) preconditioned hearts produced aortic output during reperfusion, compared to 3/6 in the non-preconditioned series.

However, in the case of hearts from young DIO rats, preconditioning did not cause a significant increase in functional performance during reperfusion and similar values were obtained as in the non-preconditioned hearts (Table 4).

The effect of diet on the preconditioning potential of hearts from middle-aged rats is summarized in Table 5. As observed previously, baseline functional performance did not differ significantly between the two DIO groups, but was significantly lower in the preconditioned age -matched control group than that of the corresponding NPC group. As in hearts from the young group, a significant reduction in aortic flow, cardiac output and Total Work occurred during reperfusion of NPC and PC control and DIO middle-aged hearts.

In contrast to the young age matched control group, ischaemic preconditioning did not improve functional performance during reperfusion in both middle-aged age-matched control and DIO groups. In fact, in both control and DIO groups, function deteriorated, although not significantly, as evidenced by the reduction in percentage recovery in Total Work (controls NPC: 42.4 ± 9.2; PC 28.4 ± 12.1; DIO NPC 41.8 ± 6.6; PC 21.6 ± 7.9). The preconditioning-induced decline in function during reperfusion, was also seen in the reduction in the number of hearts producing aortic output (Controls: NPC 7/8 vs PC 2/6; DIO: NPC 8/9 vs PC 1/6).

### Echocardiography of DIO and age-matched control rats at 17 months

Echocardiographic evaluation was done in the 17 month age-matched control and DIO groups only (Table 6). Evaluation of the young rats has been previously done (see ref 38). The left ventricle internal diameter at diastole (LVIDd) and end diastolic left ventricular volume (EDV) were significantly increased in the middle-aged DIO rats. All other parameters were similar in the two groups.

**Table 6:** Echocardiography data of middle-aged control and DIO rats

	Control (4)	DIO (4)	*p*
ESV (End systolic LV volume, ml	0.15 ± 0.02	0.17 ± 0.02	NS
FS (Fractional shortening, %)	45.04 ± 1.84	49.22 ± 1.56	NS
IVSs (Intraventricular septum thickness In systole, cm)	0.34 ± 0.01	0.32 ± 0.01	NS
LVPWd (LV posterior wall thickness at diastole, cm)	0.25 ± 0.01	0.23 ± 0.01	NS
LVIDd (LV internal diameter at diastole, cm)	0.70 ± 0.02	0.79 ± 0.02	<0.003
EDV (end-diastolic LV volume, ml)	0.78 ± 0.05	1.08 ± 0.07	<0.003
IVSd (Intraventricular septum thickness at diastole, cm	0.19 ± 0.01	0.19 ± 0.01	NS

## Discussion

The hyperphagia-induced model of obesity, as originally described by Pickavance and coworkers [34], was used in this study. This diet resulted in an initial significant rise in body weight and intraperitoneal fat seen after 16 weeks on the diet. This difference was still present after 17 months, although aging per se also caused a significant increase in intraperitoneal fat in the age-matched controls. We have previously demonstrated that the diet induced insulin resistance associated with a significant rise in serum insulin but not glucose levels [38].

A number of interesting observations were made using the well-characterized isolated working perfused rat heart for evaluation of necrosis and function: (i) exposure of hearts of young rats to 35 min of regional ischaemia resulted in a significant reduction in infarct size in DIO hearts compared to their AMC (Fig. 1), confirming previous observations made in the same model [22, 23]. With increasing the age to 17 months, however, the difference between AMC and DIO hearts was no longer observed (ii) baseline functional performance of the working heart model, was similar in hearts from AMC and DIO rats and not affected by age (iii) contrary to expectations, after regional ischaemia hearts from middle-aged AMC rats recovered functionally during reperfusion as well as their younger (22 weeks) counterparts (iv) the reduction in infarct size of DIO hearts after 22 weeks, was not associated with an improvement in functional recovery, probably due to concomitant stunning (v) infarct size as well as functional recovery during reperfusion of hearts from DIO rats were not affected by age and no differences were observed in these parameters after 17 months when compared with those after 22 weeks (vi) although hearts from middle-aged AMC and DIO rats can be preconditioned, functional recovery was depressed in both middle-aged groups despite the very significant reduction in infarct size (Fig. 3).

Effect of age: It is clear from the above summary, that the results obtained in the present study differ from the generally accepted viewpoint, namely that aging has detrimental effects on the mechanical performance of the heart as well as on its response to I/R injury. The aging cardiomyocyte has been described to develop a reduction in tolerance to stress, mitochondrial function and contractile function associated with increased susceptibility to apoptosis and necrosis (for review see [39]) with ROS playing a pivotal role. Interestingly, many of the studies on the aging myocardium have been done on mouse hearts, with mice aged 12 months being regarded as “middle-aged” and those aged 24–28 months as “aged” [11]. Since rats aged 24 months have been regarded as old [1, 2], the rats used in the present study (aged 17 months), were regarded as “middle-aged” rather than aged and could account for the fact that no marked differences in baseline mechanical performance and in the response to regional ischaemia were seen in the present study, regardless of age. However, no significant increase in infarct size between young and aging cohorts has also been reported by two other groups namely Schulman [31] and Fenton and coworkers [40]. Interestingly, in both the latter studies rats aged 18–20 months [31] and 22 months [40] were regarded as “aged”.

It should be kept in mind that the mechanical performance of the working heart model was evaluated under carefully controlled baseline conditions, such as fixed pre-and afterloads, without pacing. Their response to increased workloads, which may disclose defects in performance, was not investigated in the present study and is a topic for further experiments. It should be noted that exposure to more severe ischaemic stress, namely15 min global ischaemia, indicated that hearts from middle-aged DIO rats are more susceptible to ischaemic damage than their age-matched controls (Table 3).

Effect of obesity: Since obesity-induced insulin resistance as well I/R are known to be associated with increased oxidative stress [14–16], it was expected that the combination of age and obesity should exacerbate myocardial I/R injury. However, indications are that the model of hyperphagia-induced obesity used in the present study, is not characterized by significant oxidative stress: normalized values for serum TBARS, conjugated dienes and lipid hydroperoxide were similar to those of age-matched controls [20], suggesting that this phenomenon may not play a role in the results obtained.

Thus in the working heart model subjected to regional ischaemia, functional recovery during reperfusion of DIO hearts did not differ significantly between the young and middle-aged groups, although there are indications of deterioration in the hearts of the latter groups when subjected to global ischaemia (Table 3). This possibility is supported by the echocardiographic studies done in the middle- aged group which indicated a significant increase in LVIDd and EDV (Table 6). Dilated left ventricles with preserved systolic function are indicative of early stage dilated cardiomyopathy [41]. Whether this was the case in the DIO hearts remains to be determined. Interestingly, a similar increase in LVIDd was seen in young (16–20 week) DIO rats [42]. Obesity however had no significant effect on fractional shortening. Using a similar model of obesity, Wensley and coworkers could not find any echocardiographic changes after 32 weeks on the diet [18].

The reduction in infarct size observed in young DIO rats as well as the fact that all these hearts subjected to I/R were able to produce aortic output during reperfusion suggest that the diet protect against I/R damage, confirming previous data from our laboratory [23]. These observations are also in agreement with the findings in a similar model [22] and in fructose-fed rats [43]. However, our results are in contrast with those obtained in genetic deletion models of obesity [44–46] and in diet models [24, 47–49] where infarct size was either increased or unchanged respectively. The reasons for these controversies are not clear yet but may be due to, amongst other factors, differences in experimental protocols, diet, age and species of the animals etc. In our own hands, preference was given to use of infarct size as the more robust endpoint instead of left ventricular function which can be influenced by other factors such as concomitant stunning during reperfusion.

Explanations for the beneficial effects observed of obesity and insulin resistance on the outcome of I/R remain to be established. A previous study from our laboratory on 22 week old DIO rats did not show an association between the reduction in infarct size and activation of the RISK pathway during reperfusion [23], a signalling pathway which has been shown to be activated during cardioprotection in many experimental interventions [50, 51]. As far as we know, the role of the JAK-STAT pathway has not been investigated in this regard.

Possible signalling mechanisms involved in the effects of obese-insulin resistance have been reviewed by Apaijai and coworkers [4]. Unfortunately, as was the case in the outcome of the effects of I/R, no clear-cut pattern of changes emerged. Another factor which could contribute to the many discrepancies observed, is the fact that the time of sampling for Western blotting varied from 30 min [52] to 10 min of reperfusion [23] or before induction of ischaemia [18, 22]. Clearly further studies are required.

Preconditioning: Cardioprotection in old age and obesity has become a very relevant clinical problem in view of the fact that the developed countries are faced with an increasing elderly population. With regard to cardioprotection in the aging heart, a number of comprehensive reviews recently appeared [1–4] which indicated a loss in the ability to be preconditioned in the aging heart [27, 40, 53, 54]. For example, using infarct size as endpoint, Schulman and coworkers [31] showed that hearts from rats aged 18–20 months, could not be preconditioned by either multiple episodes of I/R or pharmacologically while the blunted response in middle-aged rat hearts could be improved by an increased preconditioning stimulus. Effective preconditioning in other aging species such as the rabbit [55] has been reported, although this was attributed to the possibility that the animals used in these two studies were probably not old enough to qualify as “old”, taking their lifespan into account [47]. Another explanation for these discrepancies is the fact that many of the previous studies used isolated hearts relying on functional recovery as endpoint [27, 28], which, in contrast to infarct size reduction, is regarded as less reliable.

Although type 2 diabetes has been associated with impaired PI3kinase/Akt signalling, GLUT4 expression as well as defects in AMPK and other kinases involved in preconditioning [56], our study showed that hearts from young DIO (insulin- resistant, but not diabetic) rats appear to be preconditioned already, while hearts from middle-aged DIO rats showed a significant reduction in infarct size when preconditioned (Figs. 2 and 3). Clearly the possibility that obesity may have beneficial effects on the myocardial response to I/R injury in young animals needs to be further investigated.

The very significant reduction in infarct size induced by ischaemic preconditioning in hearts from both middle-aged control and DIO rats (Fig. 3), was associated with a marked lowering in the ability to recover functionally during reperfusion as indicated by the overall reduction in % recovery in Total Work performed (see Table 5), when compared to their NPC counterparts. A lack of association between infarct size reduction and improved functional recovery has been observed by several groups [57–59] and attributed to concomitant stunning. It is generally accepted that myocardial stunning is due to the formation of reactive oxygen species and cytosolic calcium overloading during reperfusion (for review see [60]). Using cardiomyocytes isolated from hearts of young and aged rats, O’Brien and coworkers [53] demonstrated that although the degree of contractile depression was comparable in both age groups, the aged cardiomyocytes exhibited a much greater and more prolonged accumulation of diastolic calcium in ischaemia. Increased cytosolic calcium as well as increased oxidative stress during early reperfusion, particularly in hearts from DIO rats, may cause severe stunning in the aged hearts. In fact, it has been shown that postischaemic stunning may be exacerbated in the senescent rat heart [61]. However, despite these possibilities, functional recovery by hearts from AMC and DIO rats albeit depressed, did not differ significantly and it is only when preconditioned, that hearts from middle-aged rats showed such a marked inability to recover function. A possible explanation could be that the additional generation of ROS during the preconditioning protocol could cause the severe mechanical dysfunction in the older animals. However, in view of the many complexities of ischaemic preconditioning this phenomenon should be further investigated. In a study of potential molecular mechanisms, Griecsova and coworkers [54] demonstrated that loss of preconditioning protection was associated with age-dependent reduced Akt/PKB phosphorylation and eNOS and PKCepsilon levels in hearts of mature rats compared with the younger ones, as well as a failure of preconditioning to upregulate these proteins, confirming data obtained in an earlier study by Iemitsu et al. [62]. The involvement of the RISK pathway, particularly in the middle-aged AMC and DIO rats, in the myocardial response to I/R injury, should be further investigated, with a specific focus on the time of sampling.

## Conclusions

The data obtained in this study showed that, contrary to expectations, *middle-age as well as obesity* per se did not affect myocardial function at baseline level and did not increase the susceptibility of the heart to I/R damage, as indicated by infarct size and functional recovery during reperfusion after regional ischaemia. In fact, obesity appears to be beneficial in young animals as indicated by the reduction in infarct size when compared to their age-matched controls. These results are in contrast with the many well-established age-related modifications in the cardiovascular system [1–3], but it should be kept in mind that our observations were made in healthy animals, without the comorbidities which may affect the outcome. There is also continued interest in the ability of cardioprotective interventions such as pre-or postconditioning to protect the ageing myocardium [1–4]. Our data showed that although aging hearts exhibited normal function under baseline conditions, with an unchanged response to I/R and retention of the capacity to reduce infarct size when preconditioned, this was associated with a reduction in the ability to recover functionality. This is an important observation in view of the increasingly aging population in some countries where a loss of endogenous protection could have serious consequences for old patients. It is clear that further work is required to elucidate the effects of age and obesity on the outcome of cardioprotective interventions, with particular attention to the molecular mechanisms involved.

## Abbreviations

AMC: Age-matched controls
AMPK: AMP kinase
ANOVA: Analysis of variance
DIO: Diet-induced obesity
EDV: End diastolic left ventricular volume
EF: Ejection fraction
eNOS: endothelial nitric oxide synthase
ERK: Extracellular regulated kinase
ESV: End systolic left ventricular volume
FS: Fractional shortening
GSK-3b: Glycogen synthase kinase-3b
HSP: Heat shock protein
I/R: Ischaemia/reperfusion
IVSd: Intraventricular septum thickness at diastole
IVSs: Intraventricular septum thickness at systole
LV: Left ventricular
LVIDd: Left ventricular internal diameter at diastole
LVPWd: Left ventricular posterior wall thickness at diastole
NPC: Non-preconditioned
PC: Preconditioned
PKB: Protein kinase B
PKC: Protein kinase C
ROS: Reactive oxygen species
SEM: Standard error of mean
